# Study on Three-Dimensional Digital Expression and Robot Bending Method of Orthodontic Archwire

**DOI:** 10.1155/2018/2176478

**Published:** 2018-12-09

**Authors:** Jingang Jiang, Xuefeng Ma, Yongde Zhang, Biao Huo, Yi Liu

**Affiliations:** ^1^Robotics and its Engineering Research Center, Harbin University of Science and Technology, Harbin 150080, China; ^2^Intelligent Machine Institute, Harbin University of Science and Technology, Harbin 150080, China; ^3^Peking University School of Stomatology, Beijing 100081, China

## Abstract

Malocclusion is the third largest oral disease in the world. At present, the most effective treatment method for malocclusion is the fixed orthodontic technique based on orthodontic archwires. Robotic archwire bending can overcome the shortcomings of manual bending such as low efficiency and low precision. The three-dimensional digital expression and robot bending method of orthodontic archwire are studied to realize the orthodontic archwire bending using a robot. Tooth is identified by the doctors' common method. The shape, position, and constraint relationship of orthodontic archwire in three-dimensional space are expressed by the Bessel curve. The bending of the archwire curve is realized by transmitting the archwire curve into the alternative lines. The planning method of forming points and the spatial angle planning method are proposed. The archwire bending experiment is carried out with the maxillary information of a patient. The error rate of the experimental and ideal values is between 2.94% and 6.74%. It can meet the physician's basic requirements after simple modification. Therefore, it can be considered that the method of using discrete Bessel curve to carry out the control point planning and angle planning is suitable for the orthodontic archwire-bending robot system, which has certain feasibility and practicability in clinical treatment.

## 1. Introduction

Malocclusion is regarded as one of the three oral diseases according to the research of the World Health Organization (WHO) [1, 2]. It is always associated with teeth malposition and abnormal arrangement of the dental arch. It can affect the function of mastication and pronunciation and may easily cause dental caries, periodontitis, and other oral diseases. It also has a high incidence rate among young children [3, 4]. At present, the fixed orthodontic technique is the most effective manner to treat malocclusion. In this therapy, the deformed teeth are corrected by restoring the force that is generated by the deformation of the archwire [5].

In the process of manual archwire bending, the quality of archwire bending depends on the doctor's archwire bending skill [6]. And the treatment of different cases needs archwires with different parameters. In the bending process, archwire needs to be bent many times to realize the best therapeutic effect. Therefore, the bending efficiency is low, and the fatigue damage is more easily to produce on the archwire. [7, 8] The application of robot technology to the bending of orthodontic archwires can effectively overcome these shortcomings [9]. The bending algorithm can highly affect the bending efficiency and forming accuracy of the custom archwire. Its wide application includes the craniofacial surgery [10]. It is meaningful to study the accurate three-dimensional digital expression of orthodontic archwire as the fundamental research of the bending algorithm [11, 12].

At present, the most mature archwire bending robot in the world is the SureSmile system in the United States. It has multifunction which combines mouth scanning and archwire bending. [13] Smith et al. designed the archwire for tooth tilt correction and evaluated the experimental results with this system [14]. Gilbert developed an orthodontic archwire planning system for lingual orthodontics, which can be used to design and bend orthodontic archwire in two-dimensional plane. Hu et al. proposed the expression method of the geometric shape of the orthodontic archwire with the parameters of arch length, arch depth, and arch width [15]. Zhang compared the advantages and disadvantages of the mathematical method and finite element analysis method to describe the classical arch curve [16]. Through the linear cutting and fitting of the three-dimensional model of the orthodontic bow thread, Zhang developed the archwire-bending system including a bending software on the basis of linear cutting and fitting on the three-dimensional model of orthodontic archwire [17]. At present, the orthodontic archwire bending based on robot technology is mostly carried out in a two-dimensional plane [18, 19]. These kinds of orthodontic archwire bending robot can only bend the archwires with simple shapes, and their bending accuracy is restricted. However, there are few researches on the establishment of personalized three-dimensional model with complex curve shape. The bending algorithm is mainly divided into two parts: the planning of the forming points and the spatial angle planning of the archwire. Bending algorithm combined with the appropriate three-dimensional model of orthodontic archwire can provide an effective control strategy for the robotic orthodontic archwire bending [20].

Based on the characteristics of robotic archwire bending, this paper studies the three-dimensional digital expression of orthodontic archwire and proposes the bending algorithm based on control point planning and angle planning to ensure the efficiency and precision of robotic orthodontic archwire bending. The maxillary three-dimensional node information of a patient was used in the experiment of robotic orthodontic archwire bending.

## 2. Methods

### 2.1. Three-Dimensional Digital Expression of Orthodontic Archwire

The three-dimensional digital expression of orthodontic archwire and quantification of the shape, position, and constraint relationship need to be studied for the robotic archwire bending. In the orthodontic treatment, the orthodontic archwire needs to be connected with the bracket fixed to the tooth. According to the characteristics of orthodontic archwire positioning, the outline of the archwire can be divided into the space line of the bracket section, the spatial curve of the bracket connection part, and the spatial curve of the functional curve. The three-dimensional digital expression of orthodontic archwire can be completed by extracting the characteristics of each part to find and combine the appropriate expression mode. The expression of spatial curve is the most important step.

**Figure 1:**
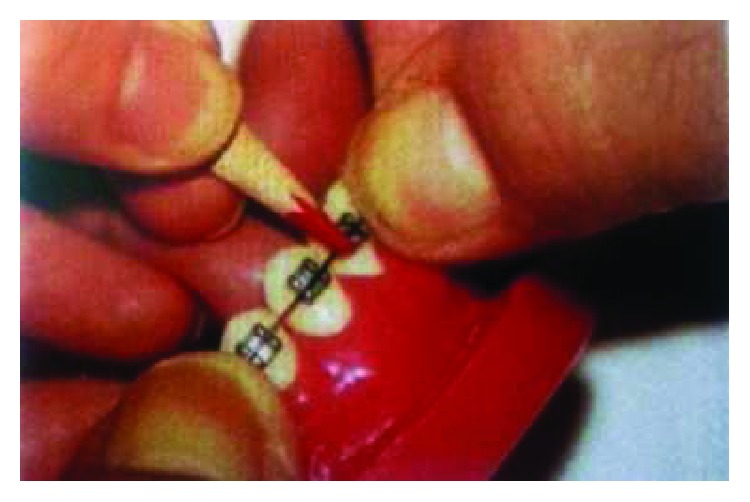
Mark of orthodontic archwire bending.

**Figure 2:**
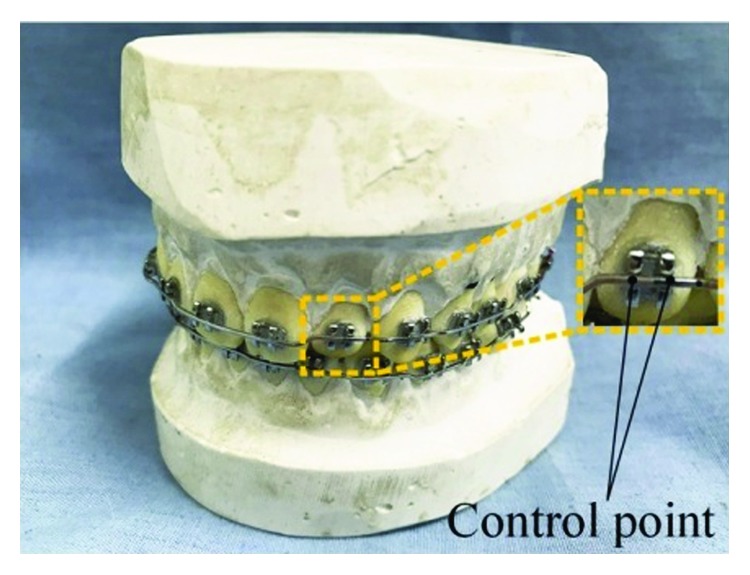
Orthodontic archwire forming reference point.

**Figure 3:**
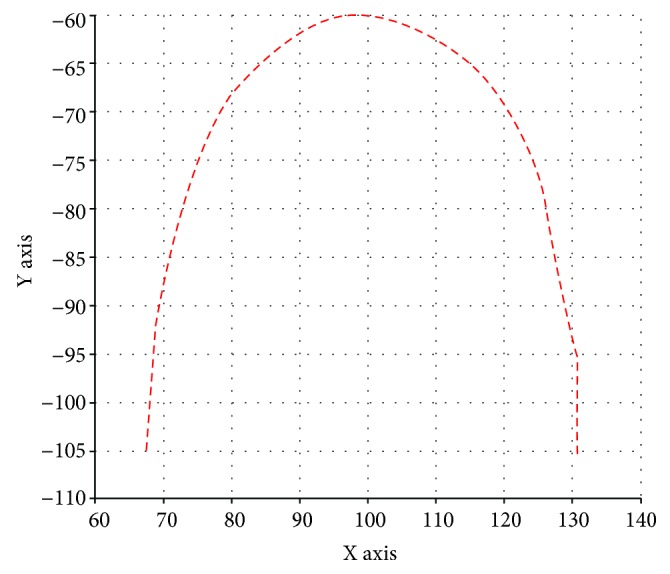
Expression of the teeth mark.

**Figure 4:**
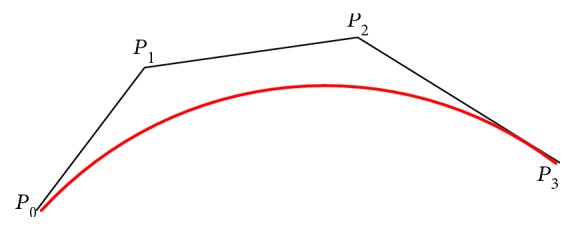
Cubic Bessel curve and the characteristic polygon.

During the process of orthodontic archwire bending, the orthodontist usually marks the position of the straight segment in bracket groove by extending a section on both sides of the bracket, as shown in Figure 1. The end points of the bracket groove are selected as the identification points of the tooth position, as shown in Figure 2. The straight line segments are obtained by connecting a pair of identification points of the tooth position, as shown in Figure 3. The space curve of the orthodontic archwire is expressed by the common curve in computer graphics. According to the forming characteristics of the orthodontic archwire, it can be found that the shape of a space curve should be controlled by a few control points, and the two tooth position control points are the start control point and the end control point. The space curve is expressed by the cubic Bezier because the archwire curve should be smooth, and the shape of the space curve can be adjusted by the control points and a series of parameters, as shown in Figure 4.

The Bezier curve is a polynomial curve uniquely defined by a set of control points. Each point can be obtained by weighted calculation of the control point coordinates. The weighted values are determined by the value *t* and BEN_*i*,*n*_(*t*), the space coordinate parameter equation is as follows:

(1) $$\begin{array}{c} \left.\begin{array}{c} P\left(t\right)=\overset{n}{\underset{i=0}{\sum }}P_{i}{BEN}_{i,n}\left(t\right),\\ {BEN}_{i,n}\left(t\right)=C_{n}^{i}t^{i}{\left(1-t\right)}^{n-i}=\frac{n!}{i!\left(n-i\right)!}t^{i}{\left(1-t\right)}^{n-i}. \end{array}\right\} \end{array}$$

In the equation, *t* ∈ [0, 1], *P*_*i*_(*x*_*i*_, *y*_*i*_, *z*_*i*_) is the position vector of each control point and *i* is the sequence number of the control point.

At least four control points *P*_0_, *P*_1_, *P*_2_, and *P*_3_ are required in the establishment of a three-dimensional archwire model. The Bessel curve can be simplified as (2) after substituting these control points into (1).

(2) $$\begin{array}{c} P\left(t\right)=P_{0}{\left(1-t\right)}^{3}+3P_{1}t{\left(1-t\right)}^{2}+3P_{2}t^{2}\left(1-t\right)+P_{3}t^{3}. \end{array}$$

The coordinates of the start control point *P*_0_ and the end control point *P*_3_ are obtained by the tooth position identification. The values of the two intermediate control points *P*_1_ and *P*_2_ are obtained by the projection distribution method. The intersection point *N*(*x*, *y*, *z*) of two adjacent teeth segments is obtained by simultaneous equations. Supposing *A*_2_ and *B*_1_ are the identification points of the tooth position of two adjacent teeth in clockwise direction, the length of the connected line is distributed according to the ratio coefficient *r* after connecting *A*_2_*N* and *NB*_1_. The coordinates of the distribution points are the control points *P*_1_ and *P*_2_. The expression of *P*_1_ and *P*_2_ are

(3) $$\begin{array}{c} \left.\begin{array}{c} P_{2}\left(x_{2},y_{2},z_{2}\right)=A_{2}\left(x_{2},y_{2},z_{2}\right)+r_{3}\cdot \vec{a},\\ P_{3}\left(x_{3},y_{3},z_{3}\right)=B_{1}\left(x_{1},y_{1},z_{1}\right)-r_{4}\cdot \vec{b}. \end{array}\right\} \end{array}$$

In the equation, *r*_3_ and *r*_4_ are the ratio coefficients; $\vec{a}$ and $\vec{b}$ are the unit vectors in the *A*_2_*N* and *NB*_1_ directions, respectively.

**Figure 5:**
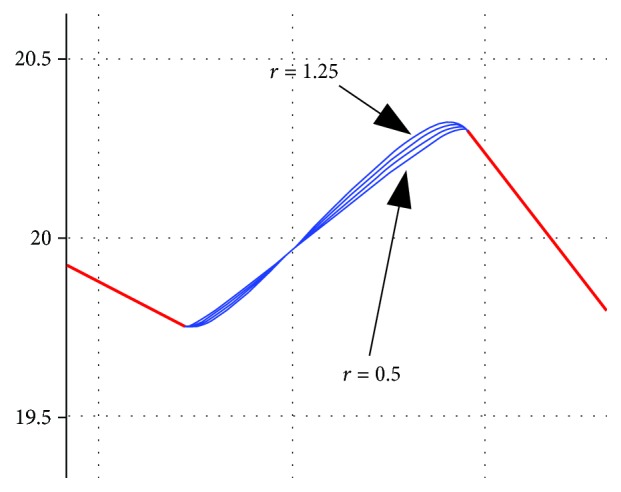
Simulation results of projection allocation method.

The result of MATLAB simulation with this method is shown in Figure 5. The curve transition is smooth when the ratio coefficient *r* is different, and the larger the *r* value is, the more obvious the convex hull is. The times of *t* taken in the range of [0, 1] is proportional to the distribution of *P*(*t*) on the curve. The smoothness of curve is also proportional to the times of *t* taken in the range of [0, 1]. Thus, the relationship between the three can be used in the modeling of the archwire. The upper and lower jaws of each person have 14 teeth, so the upper and lower arch curves should be composed of 13 sections defined by the Bessel curve and 14 sections defined by the tooth position identification. The three-dimensional curve model of the archwire is obtained by connecting these sections.

**Figure 6:**
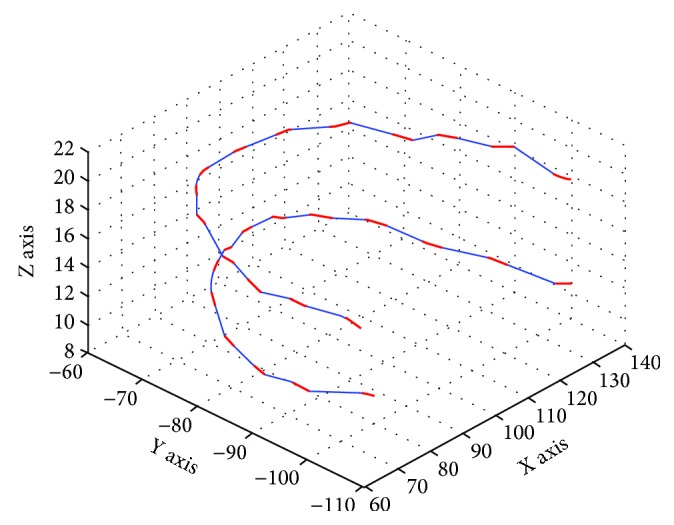
Position relationship of orthodontic archwire in patients with upper and lower jaw and the unit of length in this figure is millimeter.

This method is verified by selecting the upper and lower arch coordinates of a patient, and the ratio coefficient *r* is 0.5. The three-dimensional simulation model of the upper and lower archwires based on the Bessel curve is shown in Figure 6. The thick line in this figure represents the part of the straight lines of archwire in the brackets, and the fine line represents the Bezier curve connection lines. The results in this figure show that the Bezier connection lines and the straight lines in the brackets are connected smoothly. The overall space position and the shape of the orthodontic archwire are basically in accordance with the actual arch shape, which can be used in the archwire-bending research. Therefore, the expression method is reasonable and practical.

### 2.2. Study on Bending Method of Orthodontic Archwire Robot

The Cartesian orthodontic archwire bending robot system manufactured by Harbin University of Science and Technology is used in this study. The working space of this system is in the Cartesian coordinate system. The precision of straight line forming of this system is high and the error of curve forming is large. Thus, the archwire bending is realized by the combined movement and independent movement in the *X*, *Y*, *Z* axis direction. The continuous state is expressed by discrete motion with the method of interpolation. In the bending process, the smooth curve is represented by connecting the subdivision straight lines.

**Figure 7:**
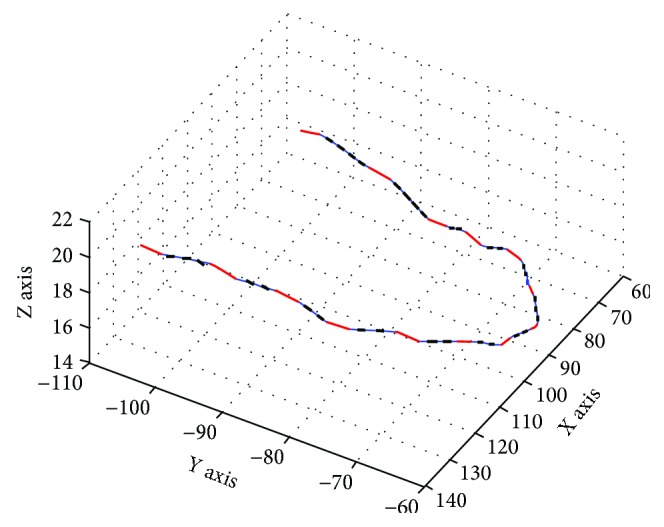
Dimensional model of orthodontic archwire curve and the unit of length in this figure is millimeter.

The bending process of the system is stable and the bending precision is fixed. According to the bending mechanism, the number of interpolation points of any curve is limited. Therefore, a curve can be represented by some straight lines with the method of interpolation. In this method, the curve is divided into some curve segments according to the interpolation points. The represented straight lines are obtained by connecting the end points of the curve segments. The expression of the complete curve expression is shown in Figure 7. In this figure, the long wide line represents the straight lines of the archwire in brackets. The long fine lines between two long wide lines are the actual space curve, and the short wide lines overlapped on the long fine lines are the interpolation lines that replace the actual curve.

#### 2.2.1. Control Planning of Bending Form Point

In the control planning of forming points, the length and the position of the start points are determined by the position and the shape of each functional curve. The number of interpolation lines which are used to replace the archwire curve is called subdivision value. For a given archwire curve, the subdivision value is in proportion to the precision of archwire bending and the similarity degree between the Bezier curve and the connected curve. The subdivision value is also in inverse proportion to the length of subdivision straight lines.

**Figure 8:**
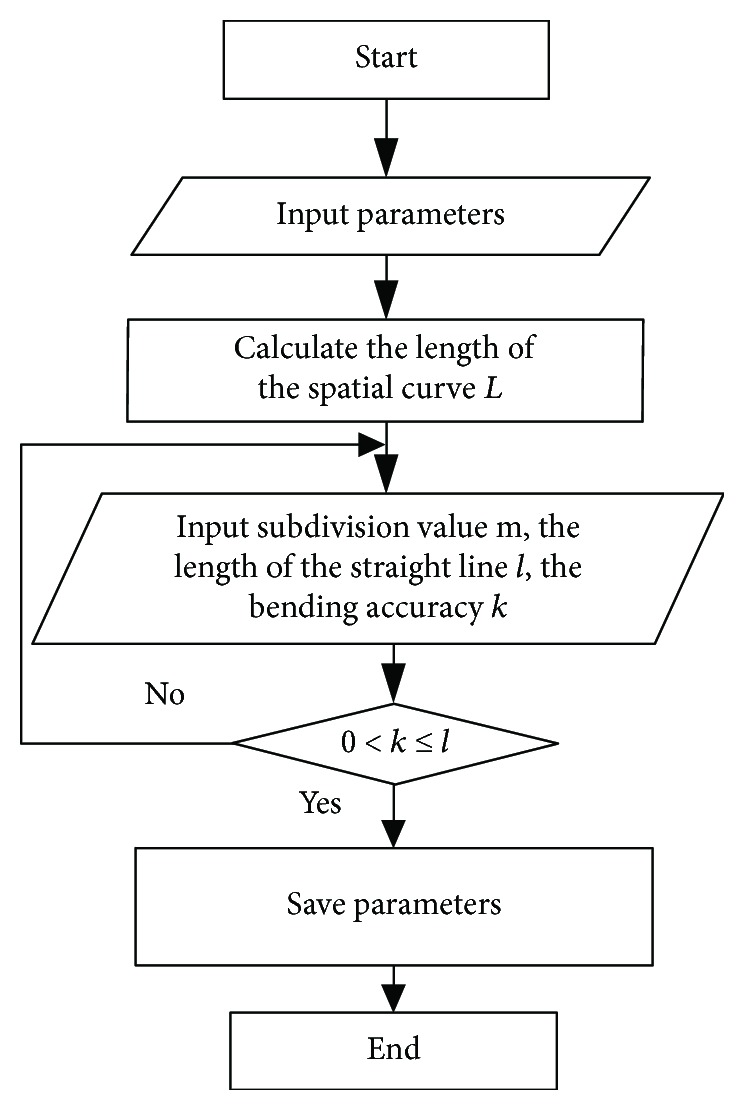
Program flow chart of bending forming point.

In the finite point generating method, the control points can be obtained by searching the limited key points. The key points of the orthodontic archwire curve are the two identification points of each teeth. The control points are the discrete forming points. The planning flow chart is shown in Figure 8, and the specific steps are as follows:

1. *Set Up the Curve Model*. The Bezier model of nonfunctional archwire curve and the functional archwire curve are established by the four control points of each curve. The three-dimensional model of the archwire curve can be obtained by connecting the nonfunctional archwire curve and the functional archwire curve in turn.
2. *Calculate the Length of the Spatial Curve*. Discrete points are the forming points which can be obtained by the discrete Bezier curve. The interpolation lines which can replace the Bezier curve are obtained by connecting the discrete point *q*_*j*_(*x*_*j*_, *y*_*j*_, *z*_*j*_)(*j* = 1, 2,…, *n*) sequentially. The length of the spatial curve is approximately equal to the sum of the length of *n* − 1 interpolation lines.
3. *The Initial Value Is Set*. The subdivision value *m* and the length *l* of the straight lines with equal length are set according to the length of the archwire curve and the smoothness of the curve after connecting the straight lines. Thus, *L* = *m* · *l*. And the bending accuracy of the robot *k* is set.
4. *The Magnitude oflandkAre Compared*. When 0 < *l* < *k*, the accuracy of the robot cannot meet the requirement of the archwire bending with a set length; this program should turn to step (3) and reset the initial value. When 0 < *k* < *l*, the accuracy of robot motion can ensure the accurate archwire bending with a set length.
5. The subdivision value *m*, the length *l* of straight line with equal length, bending accuracy *k*, and total length *L* are stored.
6. End the procedure.

In this paper, the influence factors of forming point planning, *m* and *l*, are studied instead of the influence of robot performance. An ordinary case is chosen as the research object to determine the length of the arc curve and the relationship between the length of each subdivision curve and the corresponding straight line. The planning error of each planning points is analyzed.

The control points of the second and third molars of the maxillary on the right side are as shown in Table 1. The experiment was carried out by setting the subdivision value *m* as 2, 3, and 5, respectively, *l*, the corresponding arc length $\overset{⌢}{l}$, difference value $l^{\prime }=\overset{⌢}{l}-l$, and error rate $e=\left(\overset{⌢}{l}-l\right)\times 100\%/l$. And the error results are shown in Table 2.

The subdivision value *m* is proportional to the smoothness of the straight line connection and the accuracy of the length of the archwire curve and the archwire curve segment. The subdivision value *m* is in inverse proportion to the error rate of each curve segment and the difference from the Bezier curve. The error rate of each segment is *e* < 0.38846% and the total length is *e* < 0.22801%. The error rate can meet the practical requirements.

#### 2.2.2. The Angle Planning of Curved Forming Point

The change of the angle at the forming point is also an important factor that affects the forming of the archwire. The two straight lines intersecting at a curved forming point are the two sides of the archwire angle. The forming point is the vertex of the archwire angle. Every basic rotation movement of the Cartesian robot is in the plane, so it is necessary to synthesize the basic bending angle of the robot in the *X* plane, *Y* plane, and *Z* plane.

Supposing the three bending points of the adjacent straight lines are *q*_1_(*x*_1_, *y*_1_, *z*_1_), *q*_2_(*x*_2_, *y*_2_, *z*_2_), and *q*_3_(*x*_3_, *y*_3_, *z*_3_), *q*_2_ is the vertex of the angle which is generated by the intersecting of two adjacent straight lines. Then, the vector of each straight line is

(4) $$\begin{array}{c} \left.\begin{array}{c} \vec{q_{1}q_{2}}=\left(x_{1}-x_{2},y_{1}-y_{2},z_{1}-z_{2}\right),\\ \vec{q_{2}q_{3}}=\left(x_{2}-x_{3},y_{2}-y_{3},z_{2}-z_{3}\right). \end{array}\right\} \end{array}$$

The spatial angle *θ* of the archwire at *q*_2_ is

(5) $$\begin{array}{c} \theta =\arccos\frac{\vec{q_{1}q_{2}}\times \vec{q_{2}q_{3}}}{\left|\vec{q_{1}q_{2}}\right|\times \left|\vec{q_{2}q_{3}}\right|}. \end{array}$$

**Figure 9:**
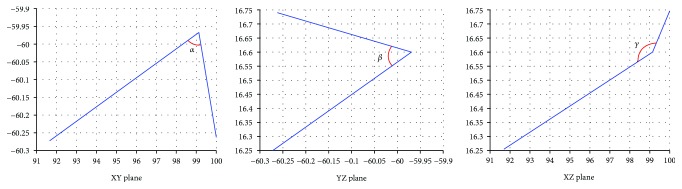
Two adjacent segments of the projective space and the unit of length in this figure is millimeter.

The spatial angle can be obtained by the corresponding angle on the projection plane. As shown in Figure 9, the included angles *α*, *β*, and *γ* can be obtained by projecting the two adjacent straight lines to the *XY* plane, *YZ* plane, and *XZ* plane, respectively. The included angle on the projection plane can be obtained from the vector angle of the projection point on each surface.

**Figure 10:**
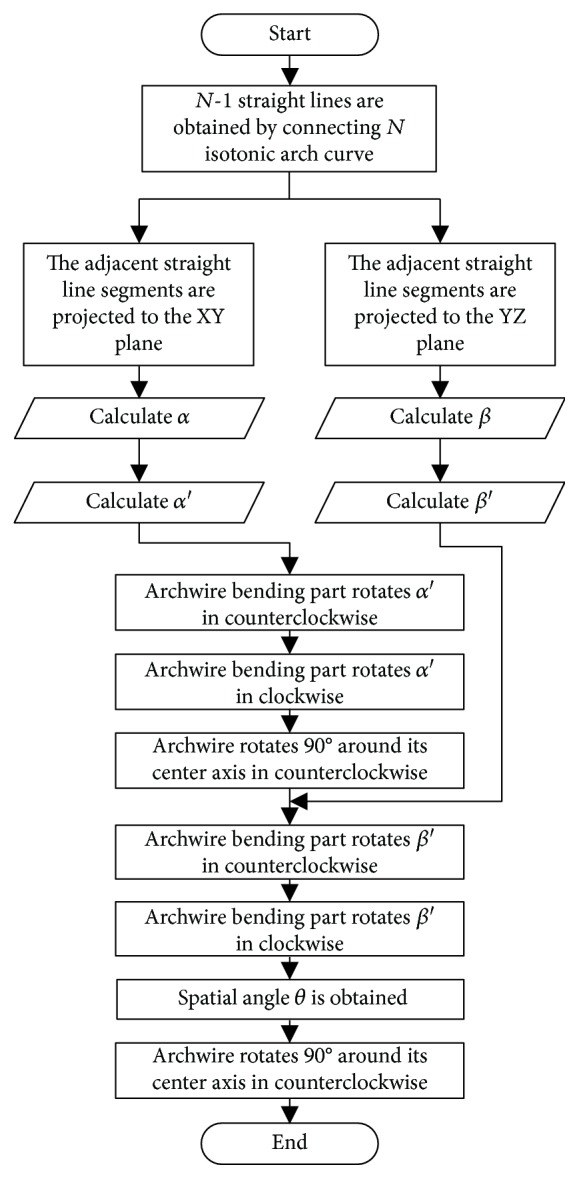
Program flow chart of angle planning on control node.

According to the geometric relation between the space angle and the corresponding projection plane angle, the included angles in the three-dimensional space of the archwire can be synthesized by the projection angle of any two projection planes. *α* and *β* are listed as the basic parameters of *θ* in this paper. The calculation process of *θ* is shown in Figure 10.

1. The adjacent straight line segments are projected to the *XY* plane, and the projection angle *α* is obtained by the vector method
2. The springback margin is studied to find the practical bending angle *α*′ after the springback from the angle *α*
3. The rotation of *α*′ angle of the archwire bending part in counterclockwise is driven by the bending mould
4. The bending mould is reset by the rotation of angle *α*′ in clockwise
5. The rotation of 90° of the archwire around its center axis is driven by the chuck
6. The adjacent straight line segments are projected to the *YZ* plane, and the projection angle *β* is obtained by the vector method
7. The springback margin is studied to find the practical bending angle *β*′ after the springback from the angle *β*
8. The rotation of *β*′ angle of the archwire bending part in counterclockwise is driven by the bending mould
9. The bending mould is reset by the rotation of angle *β*′ in clockwise
10. The archwire is reset by the rotation of 90° of the archwire around its center axis which is driven by the chuck

Thus, the spatial angle *θ* can be obtained by performing the above steps.

#### 2.2.3. Planning of Orthodontic Archwire Bending

**Figure 11:**
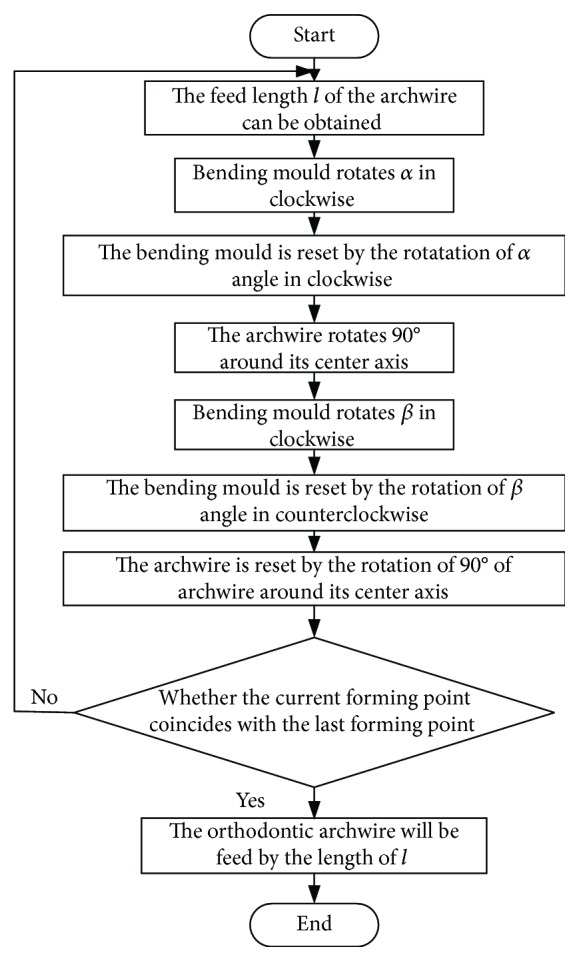
Planning flow chart of bending process.

In the archwire-bending process, the length of two forming points is controlled by the axial feed of orthodontic archwire which is driven by the electric machinery. The ideal bending angle is bent by the rotation of the archwire which goes through the bending mould. And the rotation of the archwire is driven by the bending mould. The chuck drives the orthodontic archwire to rotate around the feed axis. The bending plane is transformed by the rotation around the center axis which is driven by the chuck. And the bending of the space angle can be realized by the bending plane transformation. The bending process of robotic archwire bending can be obtained by considering the bending movement of the archwire-bending robot, the planning of forming points, and the planning of the bending angle at the forming point; the bending process is shown in Figure 11.

1. The feed length *l* of the archwire can be obtained by planning the distance between the two adjacent forming points
2. The bending movement of the angle *α* and *β* should be carried out in turn at the forming points according to the angle planning result
3. The chuck drives the archwire to rotate back to its initial position when the angle bending at archwire forming point is finished
4. Judging whether the current forming point coincides with the last forming point in the planning result, if not, then turn the program to step (1) to carry out the bending movement at the next forming point. If it coincides with the last forming point, turn the program to step (5)
5. The orthodontic archwire will be fed by the length of *l*
6. End the program

## 3. Results and Discussion

### 3.1. Selection of Experimental Use Cases

**Figure 12:**
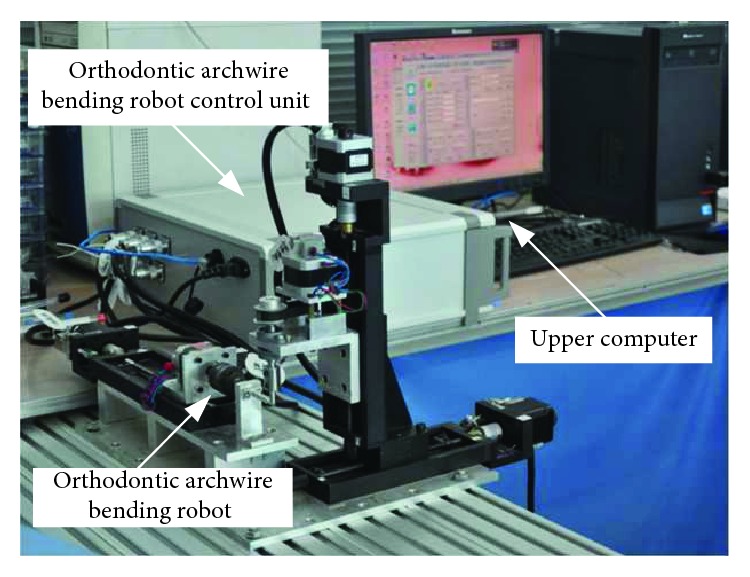
Experimental platform of orthodontic archwire bending.

**Figure 13:**
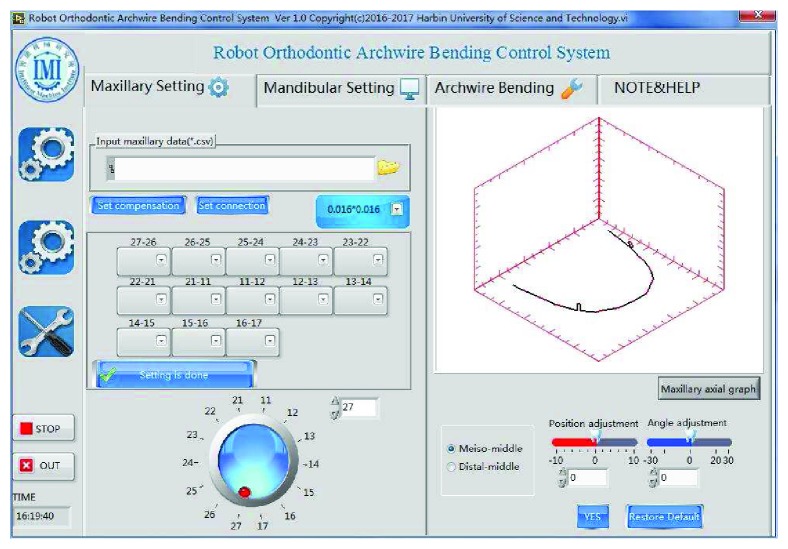
Operating interface of personalized orthodontic archwire.

In this paper, the archwire-bending experiment is carried out based on a set of three-dimensional node maxillary information of a patient, as shown in Table 3. The stainless steel rectangular archwires were used as the experimental material, and the orthodontic archwire bending robot system of Harbin University of Science and Technology was used as the experimental platform, as shown in Figure 12. Based on the above mathematical models and methods, the LabVIEW software platform is used to realize the parameterized expression and interactive adjustment method of orthodontic archwire curve. The operation interface is shown in Figure 13. With the implementation of this software, the orthodontic archwire was designed virtually in the first step. According to the designed archwire in the virtual environment, the movement of the bending robot is generated through the generation algorithm. The bending robot is directed by the bending movement to bend the archwire automatically. The important parameters of archwire forming between the results of robot bending and the result of three-dimensional digital expression were compared to verify the feasibility and accuracy of the robot bending algorithm proposed in this paper.

### 3.2. The Test Results of Orthodontic Archwire Bending

**Figure 14:**
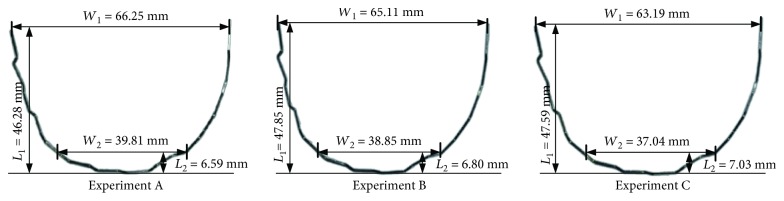
Experimental results of orthodontic archwire bending.

**Figure 15:**
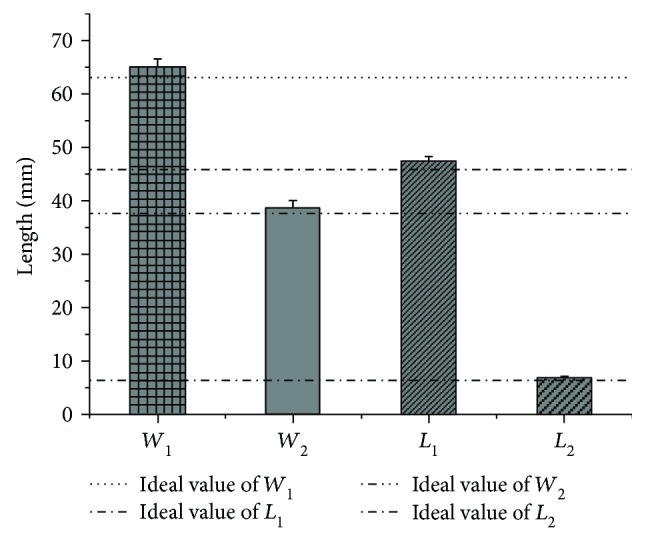
The average value of four parameters *W*_1_, *W*_2_, *L*_1_, and *L*_2_. The ideal values of these four parameters are compared with the average value of three experiments. Standard error lines shown are calculated by the results of each parameter in three experiments.

Three groups of bending experiments are carried out on the unified data considering the compensation amount of springback, and the main parameters of the experimental results are measured as shown in Figure 14. The measurement error is eliminated by measuring the average value of multiple measurements. The comparison between the average value and the ideal value is shown in Table 4. It also includes the error rate and the standard error. And the ideal values of these four parameters are compared with the average value of three experiments in Figure 15.

As shown in Table 4, the error rate of the experimental and ideal values in three experiments is between 2.94% and 6.74%. However, some limitations still existed in this study which needed to be illustrated clearly. Three orthodontic archwire-bending experiments had been conducted by the proposed orthodontic archwire-bending robot. The error rate between the ideal value and the experimental value had been calculated. The error rate can meet the requirement of the orthodontic archwire bending from the robotic consideration. At present, the orthodontic treatment is carried out by professional orthodontist. This study still needs to be verified by an orthodontist to process the further application of the robotic archwire-bending system. But from the analysis of the experiments result, it could be found that this study can meet the physician's basic requirements after simple modification. Therefore, it can be considered that the method of using discrete Bessel curve to carry out the control point planning and angle planning is suitable for the orthodontic archwire-bending robot system, which has certain feasibility and practicability in clinical treatment.

## 4. Conclusion

In this paper, a three-dimensional digital expression method based on tooth position identification was proposed for individualized orthodontic archwire. The Bessel curve was used to connect the tooth identification points. The archwire model was smooth and easy to adjust which can effectively reduce the modeling time. According to the characteristics of the archwire-bending robot structure and bending process, the robot bending algorithm based on the control point planning and angle planning was proposed. The spatial curve of orthodontic archwire model is bent and substituted by the discrete model and the method of straight line interpolation. The control point planning method based on the finite point expansion method and the angle planning method based on projection angle synthesis are proposed, which can effectively obtain the information of archwire bending control point and its spatial angle. The experimental result shows that the error rate of the main parameters of the archwire meets the clinical requirements, and the validity and feasibility of the orthodontic archwire-bending algorithm based on the robot are verified by the bending experiment of a group of randomly selected cases.

## Figures and Tables

**Table 1:** The control point information of the spatial curve between the right second molar and the third molar of the maxillary.

Object	Coordinate points
Start control point A	(130.2148, −102.7720, 19.7540)
End control point B	(130.5915, −95.4711, 20.3004)
Middle control point P1	(130.0832, −101.7821, 19.7005)
Middle control point P2	(130.8552, −96.4265, 20.4346)

**Table 2:** Length and deviation calculation for node planning.

*m*		First-section length	Second-section length	Third-section length	Fourth-section length	Fifth-section length	Total length
2	$\overset{⌢}{l}$	3.67861	3.66983	—	—	—	7.34844
	*l*	3.66432	3.66736				7.33168
	*l*′	0.01429	0.00247				0.01676
	*e*	0.38846	0.06730				0.22801

3	$\overset{⌢}{l}$	2.45219	2.44194	2.45431	—	—	7.34844
	*l*	2.44841	2.44192	2.44519			7.33554
	*l*′	0.00378	0.00002	0.00912			0.01293
	*e*	0.15415	0.00082	0.37159			0.00176

5	$\overset{⌢}{l}$	1.46908	1.46628	1.46703	1.47177	1.47428	7.34844
	*l*	1.46661	1.46627	1.46702	1.47168	1.46851	7.34009
	*l*′	0.00247	0.00001	0.00001	0.00009	0.00577	0.00835
	*e*	0.16813	0.00068	0.00060	0.00612	0.00391	0.11363

**Table 3:** Maxillary three-dimensional node information.

Object	*X* coordinate	*Y* coordinate	*Z* coordinate
1	130.6208	−105.8205	19.9202
2	130.2148	−102.7719	19.7543
3	129.5915	−95.4711	20.3004
4	129.6021	−91.8770	19.7956
5	127.7818	−86.7641	19.5864
6	127.2209	−83.3877	19.3124
7	125.7315	−79.9555	18.4712
8	124.9892	−76.7429	18.3497
9	121.6885	−70.9943	18.5183
10	119.4489	−68.4552	18.0067
11	114.4043	−64.5938	17.7725
12	111.6932	−63.5352	17.5282
13	106.1981	−61.0719	16.8799
14	103.2650	−60.8079	16.8648
15	96.6560	−60.0703	16.4737
16	93.7371	−60.1091	16.3261
17	87.8990	−63.1470	16.8093
18	85.6415	−64.4220	16.6305
19	79.5748	−68.0257	16.7521
20	77.8668	−70.6027	16.7358
21	74.8902	−75.6715	15.8080
22	73.6637	−78.2759	15.9849
23	72.2534	−81.7699	15.6494
24	71.1393	−84.7600	15.4502
25	68.9799	−91.2384	16.4979
26	68.4687	−94.5584	16.5958
27	67.9186	−102.0036	17.3374
28	67.7512	−105.9736	17.1265

**Table 4:** Comparison of experimental value with theoretical value.

Object	*W*_1_	*W*_2_	*L*_1_	*L*_2_
Experiment				
Experiment A	66.25	39.81	46.28	6.59
Experiment B	65.11	38.85	47.85	6.80
Experiment C	63.19	37.04	47.59	7.03
Average value	64.85	38.57	47.24	6.81
Ideal value	62.93	37.47	45.75	6.38
Error rate	3.05%	2.94%	3.25%	6.74%
Standard error	1.59	0.58	0.47	0.18

## Data Availability

The data used to support the findings of this study are available from the corresponding author upon request.
